# The efficacy, safety and cost-effectiveness of hydroxychloroquine, sulfasalazine, methotrexate triple therapy in preventing relapse among patients with rheumatoid arthritis achieving clinical remission or low disease activity: the study protocol of a randomized controlled clinical Trial (ESCoRT study)

**DOI:** 10.1186/s12911-021-01449-2

**Published:** 2021-03-04

**Authors:** Juan Zhao, Wei Zhou, Yangfeng Wu, Ping Ji, Li Yang, Xiaoyan Yan, Zhuoli Zhang

**Affiliations:** 1 Department of Rheumatology and Clinical Immunology, Peking University First Hospital, Beijing, 100034 China; 2 Peking University Clinical Research Institute (PUCRI), Beijing, 100083 China; 3 Peking University School of Public Health, Beijing, 100083 China

**Keywords:** Rheumatoid arthritis, Pharmaceutical economics analysis, Cost-effectiveness analysis, Cost-utility analysis, Relapse

## Abstract

**Background:**

Tumor necrosis factor α inhibitors (TNFi) is effective for rheumatoid arthritis (RA) patients who fail to conventional synthetic disease-modifying anti-rheumatic drugs (csDMARDs). Because of high cost, the discontinuation is common but often lead to disease relapse. The study aims to investigate, if the combination therapy of csDMARDs is more effective in reducing disease relapse than methotrexate (MTX) monotherapy, and more cost-effective than continuing TNFi and MTX.

**Methods:**

It will be a two-stage trial. In the first stage, all RA patients who failed to csDMARDs treatment [disease activity score 28 (DAS28)-CRP > 3.2] will receive MTX plus TNFi for no more than 12 weeks. Patients achieving DAS28-CRP < 3.2 during the first stage will be randomized into three groups at 1:1:1 ratio: (A) add hydroxychloroquine (HCQ) and sulfasalazine (SSZ) for the first 12 weeks and then remove TNFi but continue other treatments for the next 48 weeks; (B) maintain TNFi + MTX for 60 weeks; and (C) maintain TNFi + MTX for the first 12 weeks and then remove TNFi but continue MTX monotherapy for the next 48 weeks. The primary outcome will be disease relapse (DAS28-CRP increases by at least 0.6 and > 3.2). Secondary outcomes will include the incremental cost per reducing 1 case of relapse; patient reported intolerance to the treatment; adverse events; change of mean disease activity measured by DAS28, clinical disease activity index (CDAI) and simplified disease activity index (SDAI); the proportion of modified Sharp score increase < 0.3; ultrasound-detected remission in hands; Health Assessment Questionnaire Disability Index (HAQ-DI) and health related quality of life [the five-level EuroQol-5D (EQ-5D-5L) and short form-6D (SF-6D)].

**Discussion:**

The aim of this trail will be to seek effective treatment options of preventing relapse of RA. The results of the current study may provide an instructive recommendation for more economical application of TNFi treatment in RA.

*Trial registration* NCT, NCT02320630. Registered on 16 December 2014. https://register.clinicaltrials.gov/prs/app/action/LoginUser?ts=3&cx=-jg9qo2.

## Background

Rheumatoid arthritis (RA) will cause joints deformity and functional loss if not treated with early effective therapy. Only 30% to 40% of RA patients could achieve good response with the treatment of methotrexate (MTX) monotherapy [1]. The treatment effectiveness has been greatly improved by biological disease-modifying anti-rheumatic drugs (bDMARDs), especially Tumor Necrosis Factor α inhibitors (TNFi). “Treat to target” (T-to-T), achieving clinical remission (CR) or low disease activity (LDA) alternatively for long term, has become the core strategy of RA treatment. According to the most recent recommendations by both European League against Rheumatism (EULAR) and American College of Rheumatology (ACR), after the failure of conventional synthetic disease-modifying anti-rheumatic drugs (csDMARDs), the application of biological agents, especially TNFi, is recommended [2].

The discontinuation of TNFi often leads to increased relapse rate of RA. In a recently published clinical trial, early RA patients who had achieved clinical remission or low disease activity by adalimumab and MTX were randomized to maintain or discontinue adalimumab. During a 78-week observation, 81% of those who stopped adalimumab maintained LDA, comparing to 91% of those who continue to receive adalimumab [3]. It was also reported that for established RA patients who have achieved CR or LDA by TNFi combined with MTX, 24% to 85% suffered from disease relapse after discontinuation of TNFi [4–7].

However, due to the high cost, the demand for discontinuation of TNFi is common. For patients who have achieved the treatment target, strategies to balance the expense and the risk of relapse are urgently needed. It has been reported that, in RA patients who achieved CR with TNFi combining MTX, the proportion for patients who maintained CR with half-dose of TNFi was comparable to those with full dose, and both were higher than those who discontinued TNFi [8].

An increasing number of studies showed that the efficacy of csDMARDs combination therapy is superior to monotherapy [9]. The classical combination strategy of MTX plus hydroxychloroquine (HCQ) and sulafasalazine (SSZ) provided good response and safety [10, 11]. It has been reported that 77% refractory RA patients had 50 percent improvement at nine months without major drug toxicity with MTX + HCQ + SSZ triple therapy [10]. Nevertheless, the BeSt study showed that there was little difference between csDMARD monotherapy and combination therapy [12]. A previous study showed that combination therapy of MTX and cyclosporin could not prevent relapse in 58% RA patients after discontinued biological DMARD [4]. In that study, cyclosporin was just initiated at the discontinuation of TNFi. However, considering the delayed efficacy of csDMARDs, it is preferable to overlap csDMARDs and TNFi for several weeks before discontinuation of TNFi.

Long-term therapy of bDMARDs poses a heavy burden on RA patients and the society. The commonly used csDMARDs are much cheaper than biologics [13]. An investigation showed that only 9% of Chinese RA patients received biological DMARDs. However, the cost of biologics accounted for 49% of the total cost for RA treatment medications. Due to lack of high quality research, it is still under debate whether the benefit is worth such high cost.

This 1:1:1 randomized enrollment parallel group study aims to investigate, among RA patients who are refractory to csDMARDs but have achieved CR or LDA after treatment with TNFi and MTX, if the triple therapy of MTX + HCQ + SSZ is more effective in reducing the relapse rate than MTX monotherapy, and more cost-effective than continuing the treatment of TNFi and MTX.

## Methods/design

### Study design

This will be a two-stage study. The first stage will serve as an induction treatment with TNFi plus MTX for no more than 12 weeks. Patients who have achieved CR or LDA [defined as disease activity score 28 (DAS28)-CRP ≤ 3.2] in 12 weeks will enter the second stage at the time of CR/LDA achieved. The second stage will be a multiple-center, randomized, outcome assessment blinded, parallel controlled clinical trial. All eligible participants will be randomly allocated to three arms at a ratio of 1:1:1 to receive different treatment strategies for 60 weeks or until relapse.

**Fig. 1:**
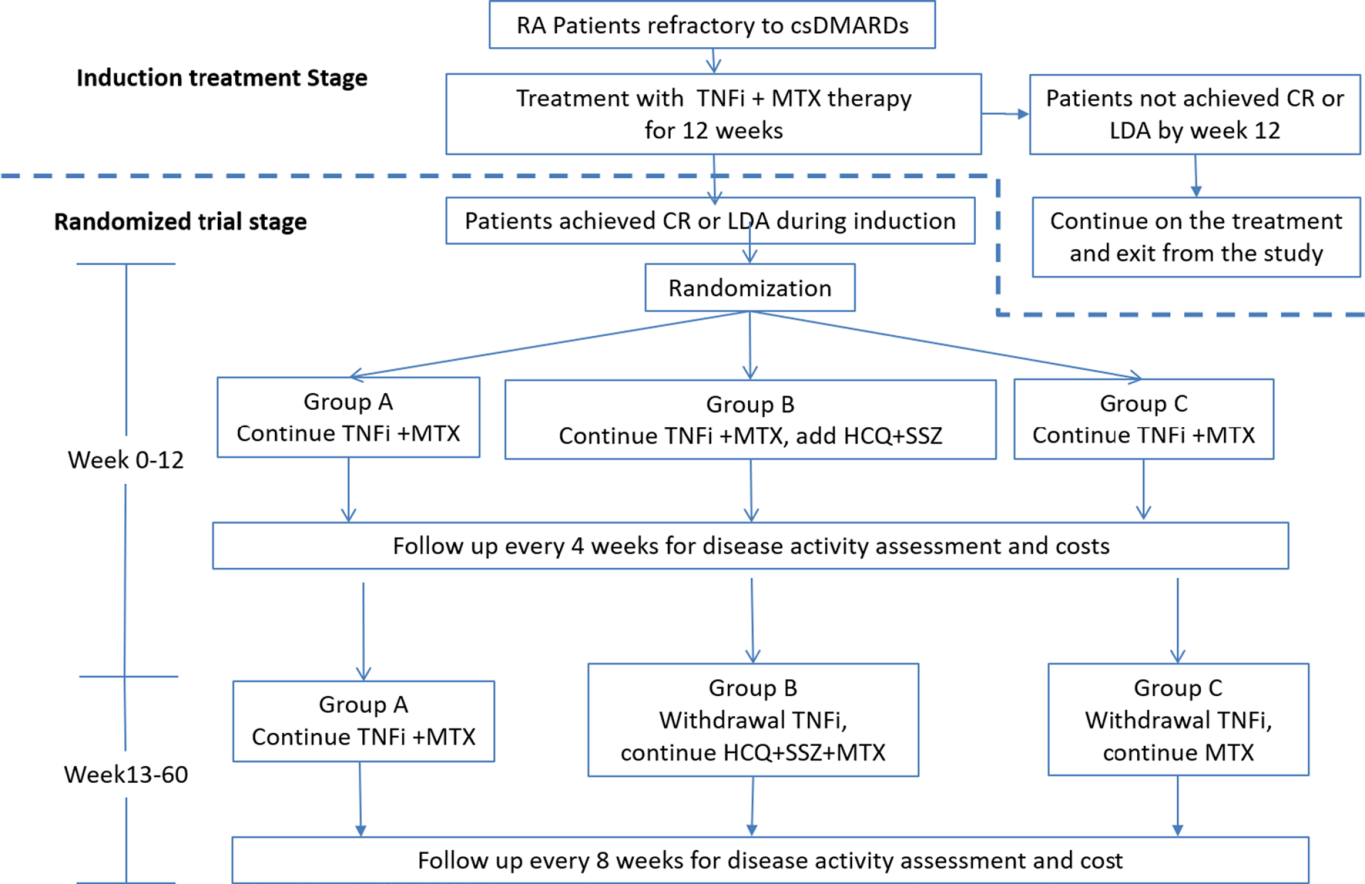
The study flow chart. Note: for patients who relapse during follow up, the study treatments will be stopped and patients will be referred to appropriate alternative treatments and follow up will be continued for cost data collection. RA: Rheumatoid Arthritis; csDMARDs: conventional synthetic disease-modifying anti-rheumatic drugs; MTX: methotrexate; HCQ: hydroxychloroquine; SSZ: sulfasalazine; TNFi: Tumor necrosis factor α inhibitors; CR:clinical remission; LDA: low disease activity

This study will be expected to be conducted from October 2015 to September 2022. The first patient was recruited on Oct 8, 2015 and the last patient is planned to be recruited by Sep 30, 2021. The study flow-chart is shown in Fig. 1.

### Eligibility criteria

1. Inclusion criteria
  1. Fulfill the 2010 EULAR/ACR diagnostic criteria of RA;
  2. Disease duration > 6 months;
  3. ≥18 and≤70 years old;
  4. Use of single or combination of csDMARDs with standard dosage (must including MTX and/or leflunomide) for more than 3 months;
  5. DAS28-CRP> 3.2;
  6. Sign informed consent form (ICF).
2. Exclusion criteria
  1. Receive therapies of any of the below:
    - Large surgical operations within 8 weeks (including articular operation) or in plan;
    - Any former use of cell elimination therapy, including CAMPATH, anti-CD4, anti-CD5, anti-CD3, anti-CDl9, anti-CD20 and the research drug in the current study;
    - γ-immunoglobulin, plasmapheresis, retuximab or IL-6 inhibitor within 6 months;
    - Any form of glucocortocoid injection therapy within 4 weeks;
    - Live vaccines or live attenuated vaccines within 4 weeks;
    - Any alkylating agents including cyclophosphamide and nitrogen mustard, or total lymphoid irradiation therapy;
  2. Complicate with any other diseases or organ/tissue damages as follows:
    - Autoimmune diseases other than RA. Patients combining with primary SjÖgren syndrome (pSS) are permitted;
    - Severe or uncontrolled cardiac disease, nervous system disease, pulmonary disease (including chronic obstructive pulmonary disease and interstitial lung disease), renal disease, liver disease, endocrine disease (including diabetes mellitus) and gastrointestinal disease;
    - Diseases that need oral or injection therapy of glucocorticoid, including but not limited to bronchial asthma, psoriasis and inflammatory bowel disease;
    - Current or relapsing infections (include but not limited to tuberculosis or atypical mycobacterium disease, granuloma in chest X ray, type B or type C hepatitis, human immuno-deficiency virus (HIV), Zoster, but patients with nail fungal infection are permitted). Infection that needs hospitalization or intravenous antibiotics within 4 weeks, or oral antibiotics within 2 weeks;
    - Malignant disease, including solid tumor and hematological malignancies (but patients with moved or cured skin basal cell carcinoma are permitted);
    - Nerve damage or other painful disease that may affect pain evaluation;
  3. Laboratory abnormalities:
    - Serum creatinine> 130umol/L;
    - Alanine aminotransferase (ALT) or aspartate transaminase (AST) > 2 upper limit of normal, or total bilirubin> upper limit of normal;
    - Platelet <100 × 109/L, or white blood cell count (WBC) <3 × 109/L;
    - Interstitial lung disease: confirmed by chest X ray;
    - Double hands X ray shows ACR imaging stage IV;
  4. Allergic to or have severe adverse reaction with any experimental drug in the induction or randomization stage;
  5. Pregnant or plan to be pregnant in 2 years, or nursing mothers.

Participants will be recruited into this study from seven participating research centers. As soon as the patients achieve DAS28-CRP ≤ 3.2 during the induction phase, they will be further recruited into the second stage of randomized phase. Patients who do not achieve clinical remission or LDA by the end of 12 weeks of the induction phase will exit the study and receive empirical treatment.

### Randomization

The blocked randomization, stratified by study center and gender of patients, will be performed centrally when CR or LDA is achieved (V0, week 0). The staffs responsible for randomization will obtain the group allocation code from the Peking University Clinical Research Institute (PUCRI) through an Interactive Web Response System (IWRS).

### Interventions

Enrolled subjects will receive oral MTX 10–20 mg once a week and subcutaneous injection of TNFi 50 mg once a week in the induction stage.

Patients who did not receive MTX before recruitment, will initiate MTX 10 mg per week at enrollment. In the induction stage, MTX will be allowed to be titrated up to 20 mg per week if the patient do not achieve DAS28-CRP ≤ 3.2.

The three treatment strategies at the randomized stage will be:

Group A: add HCQ 200 mg twice per day and SSZ 1000 mg twice per day for the first 12 weeks and then remove TNFi but continue MTX + HCQ + SSZ for the next 48 weeks (intervention group, triple therapy group);

Group B: maintain TNFi + MTX for all the next 60 weeks (control group 1, TNFi maintenance group);

Group C: maintain TNFi + MTX for the first 12 weeks and then remove TNFi but continue MTX monotherapy for the next 48 weeks (control group 2, MTX monotherapy group).

### Concomitant medications

#### Concomitant medication not permitted

Glucocorticoids and other DMARDs, including leflunomide, azathioprine, tripterygium, cyclosporine, and other biological agents.

#### Concomitant medication permitted

NSAIDs will be permitted, but the drug name, dosage and time of treatment should be recorded.

Anti-hypertension medications, anti-diabetic agents, aspirin and β-blocker will be permitted.

All the concomitant medications used during the study should be recorded in the case report form (CRF).

### Follow up schedule and data collection

A total of at most 14 visits will be scheduled. Data to be collected at each visit are summarized in Table 1.

**Table 1:** Follow up schedule and data to be collected

Study period	Induction treatment stage		Randomized stage										Early exit
Visit	S1	S2-4	V0	V1	V2	V3	V4	V5	V6	V7	V8	V9	
	Wk0	Wk 4\8\12	Wk0	Wk4	Wk8	Wk12	Wk20	Wk28	Wk36	Wk44	Wk52	Wk60	
ICF	√												
Demographic data	√												
Past history and medication history collection	√												
Physical examination	√	√ √	√	√	√	√	√	√	√	√	√	√	√
Urine βHCG (if applicable)	√												
BCC, renal function, liver function, ESR,CRP,RF	√	√	√	√	√	√	√	√	√	√	√	√	√
Assessment committee examination	√	√ √	√	√	√	√	√	√	√	√	√	√	√
IC/EC judgement	√												
Randomization		√											
Hand X-ray-ran	√		√									√	√
Ultrasound of double hands	√		√									√	√
Anti-CCP antibody	√											√	√
Cost of exam and treatment	√		√	√	√	√	√	√	√	√	√	√	√
HAQ-DI/EQ-5D	√		√			√						√	√
Study Medication record	√	√	√	√	√	√	√	√	√	√	√	√	√
Concomitant medication record	√	√ √	√	√	√	√	√	√	√	√	√	√	√
AE record	√	√ √	√	√	√	√	√	√	√	√	√	√	√

*ICF* informed consent form, *BCC* blood cells count, *ESR* erythrocyte sedimentation rate, *CRP* c reactive protein, *RF* rheumatoid factor, *IC/EC* inclusion criteria/exclusion criteria, *Anti-CCP antibody* anti-cyclocitrulline polypeptide antibody, *HAQ-DI/EQ-5D* Health Assessment Questionnaire Disability Index/ Euro Qol five dimensions questionnaire, *AE* adverse event

Patients will be requested to return the residual drugs to obtain new drugs at each visit. Laboratory tests must be performed in the eligible study centers. These strategies were designed to improve adherence of subjects.

Subjects who relapse or cannot tolerant adverse effects before the end of the trial should exit the trial and receive empirical treatment, including but not limited to, other biological agents, or csDMARDs combination therapy. All patients who exit early will be kept in following up for the safety and cost-effectiveness analysis.

### Study outcomes

The primary outcome will be disease relapse, which is defined as DAS28-CRP increases by at least 0.6 compared with that at randomization and is greater than 3.2 simultaneously.

The secondary outcomes will include the following: the incremental cost per reducing 1 case of relapse; change of disease activity score from baseline, assessed by DAS28-CRP, DAS28-ESR, CDAI and SDAI, at 60 weeks after randomization; duration of maintaining CR or LDA after randomization; change of hands X ray modified Sharp score at 60 weeks after randomization; ultrasound remission of hands at 60 weeks after randomization; Health Assessment Questionnaire Disability Index (HAQ-DI) and health related quality of life [the five-levele EuroQol-5D-5L (EQ-5D-5L) and short form-6D (SF-6D)] at 60 weeks after randomization; adverse events and complications; and patient reported outcomes (PRO) including rash, nausea, vomiting, diarrhea, abdominal pain and fever.

All outcomes will be measured after 60 weeks.

Responsible physicians will monitor and record any adverse events (AEs) during the whole trial. Expected AEs will include: allergy; ALT and AST elevation; hemocytopenia; infection; digestive tract symptom including nausea and vomiting; and hemorrhage of digestive tract defined by fecal occult blood positive, etc. In case of AEs, symptomatic treatment or reduction of drug dosage or even discontinuation of drugs will be carried out when necessary. If the drugs is tapered or discontinued, a serious AE will be considered and the patient should exit the study. Physicians will assess the association between intervention and AEs on a 6-point scale (1 = definitely related; 2 = probably related; 3 = possibly related; 4 = probably not related; 5 = definitely not related; 6 = unknown). All AEs will be categorized into three levels using the Spilker classification [(1) mild = not need additional intervention, nor significantly inhibit normal life function of the participant; (2) moderate = significantly inhibit the normal life function of the participant, and may need additional intervention, recovering afterwards; and (3) severe = require intensive intervention, and leave sequelae).

Serious adverse event (SAE) is defined as any of the following conditions: (1) threat to life; (2) lead to hospitalization or extend hospital stay; (3) lead to permanent or notable disability or functional disorder; and (4) lead to taper or discontinue the study drugs. In case of SAE or scheme violation during study duration, the relevant Institutional Review Board (IRB) and the leading study site (Peking University First Hospital) will be informed and will decide whether the trial will continue or not. Patients will be followed even if protocol has been breached for any reason. The SAE will be recorded as safety outcome.

An independent assessment committee will be set up for outcomes evaluation. The assessment committee will be blinded to the treatments. However, in case of “SAE”, unblinding will be permitted.

### Exit criteria

1. Patients or their authorized representative require withdrawal from the study;
2. Do not achieve DAS28-CRP < 3.2 by the end of 12 weeks in the induction treatment;
3. Relapse during the randomized phase;
4. Because of AE or SAE, the responsible physician considers that the patient is not suitable to continue the trial;
5. Because of protocol violation (PV), the responsible physician considers that the patient is not suitable to continue the trial;
6. Become pregnant.

Participants will be advised verbally and in writing that they will be free to withdraw at any stage upon request.

### Data management and quality control

The Data Management Department of PUCRI (PUCRI-DM) will be responsible for data management, including developing an electronic CRF and data capture system, drafting the data management plan, the data verification plan, plans for promoting participant retention and completing follow-up, and plans for data entry, coding, security and storage. Data management plan will describe details of the whole process of data management, schedule, and responsibilities of every staff. The system will support electronic signature, access control, data query and trace management. The database will be locked before transferring to the statistician for data analyses.

A special training session on the study protocol and CRF will be provided before the initiation of the study at each study site. The on-site monitoring will be conducted by the clinical researchers from the PUCRI at the beginning, in the middle and at the end of the study to ensure the study protocol is not violated.

The PUCRI will assign a project specialist to be responsible for the data monitoring. The monitor will confirm the implementation of the project, the compliance of data record and relevant regulations. The content of monitoring will include signing of ICF, incidence of SAE and PV, as well as management of study medications. After monitoring, a report will be generated and submitted to the project managers. The monitor will be independent of the investigators and sponsor.

Auditing of the trial will be conducted after complement of the study by the finance department which is independent of the investigators and the sponsor.

#### Sample size estimation

According to the study purposes, a superiority design was considered appropriate. Our hypothesis is that the relapse rate in group A (triple therapy group) will be lower than that in group C (MTX monotherapy group) in 60 weeks after randomization.

Sample size was calculated by PASS 11.0. It was reported that for patients with established RA achieving CR or LDA by TNFi combined with MTX, 24% to 85% suffered from disease relapse after discontinuation of TNFi [4–7]. When combined csDMARDs preserved after TNFi removed, the rate of relapse was 51.2–58% [4, 7]. We assume that adding HCQ + SSZ 3 months before the removal of TNFi may somehow decrease the relapse. Accordingly, we expect the relapse rate as 50% in patients in triple therapy group, and 74% in MTX monotherapy group. With a power of 0.8 and a significance level of 0.05 (two-sides), using a 1:1:1 treatment allocation of enrollment, to detect the difference of relapse rate between the two groups, the required sample size is 61 for each group. Given the estimated dropout rate of 20%, we intend to recruit a total of 240 subjects (80 for each group).

We assume that the relapse rate may be similar between group A and group B (TNFi maintenance therapy group), and the cost-effectiveness may be better in group A than in group B. The relapse rate and cost-effective comparison between group A and group B will be exploratory and there was no relevant research hypothesis, so the sample size was not calculated by it.

#### Statistical analysis

All statistical analyses will be performed using SAS 9.2.

The compatibility of baseline data among three groups will be checked. Student’s t-test or Wilcoxon rank sum test will be used for quantitative variables. Chi-square test or Fisher’s exact test will be used for categorical variables. The Cochran–Mantel– Haenszel test or the Wilcoxon rank sum test will be used for ordinal variables.

To compare the relapse rate between triple therapy group and MTX monotherapy group, the intention-to-treat principle will be followed. Considering the influence of central effect, the collected data will be estimated by mantel Haenszel method with stratification by study centers. Kaplan–Meier curves will be used to show the differences of relapse rate among three groups. The disease activity changes and other continuous secondary outcomes (Sharp score, ultrasonic Doppler score and HAQ-DI score) will be compared between the study groups using the covariance analysis model. Per protocol analyses will also be conducted as sensitivity analyses.

**Fig. 2:**
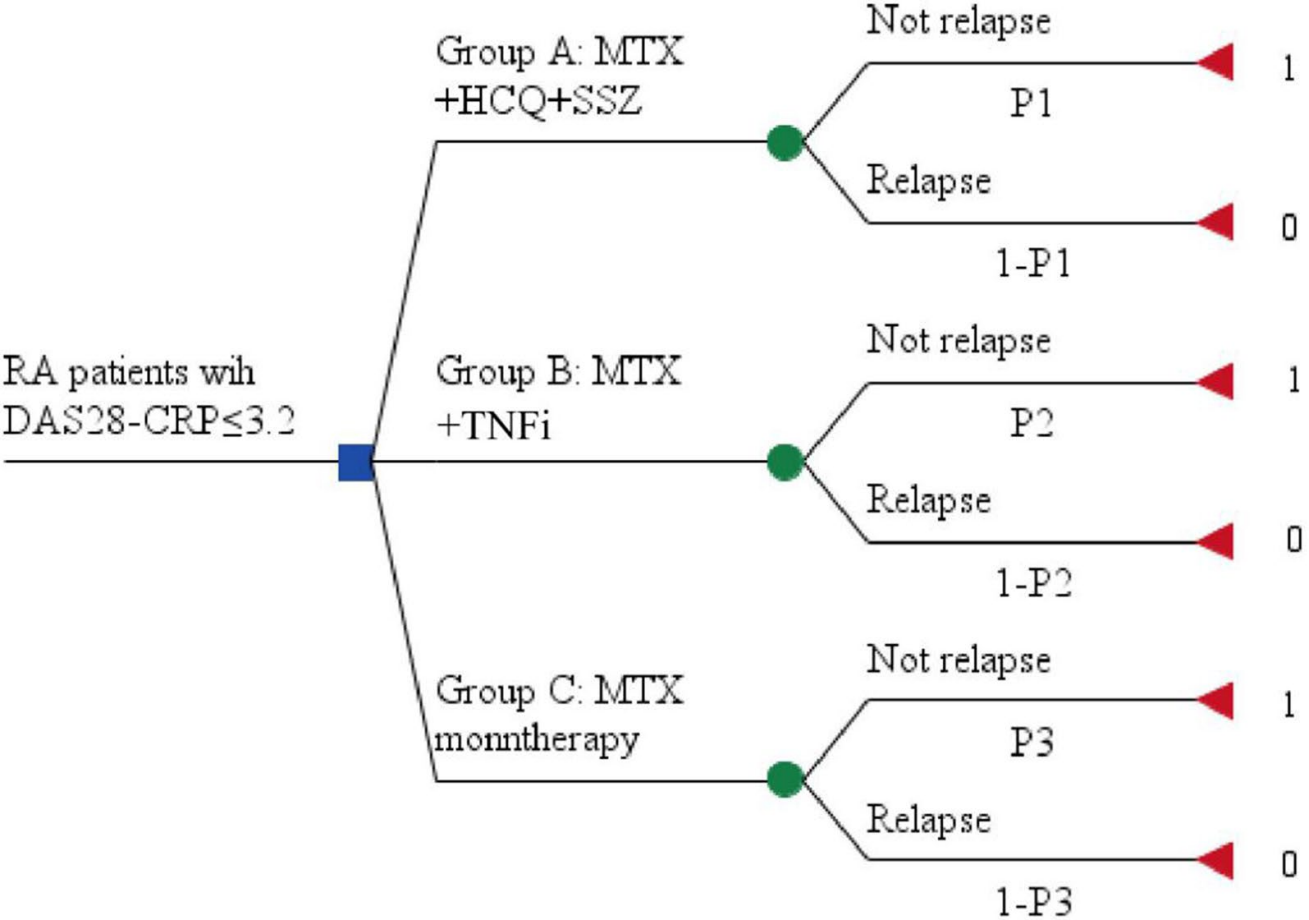
The decision tree model of the study. RA: Rheumatoid Arthritis; MTX: methotrexate; HCQ: hydroxychloroquine; SSZ: sulfasalazine

Pharmaceutical economics analysis will be performed. Base case analysis and sensitivity analysis will be performed by decision tree model based on Per-Protocol Set (Fig. 2). A series of one-way deterministic sensitivity analysis will be conducted. For the cost-effectiveness analysis, the proportion of not relapse will be taken as effectiveness. The direct cost (medical expenses), indirect cost (productivity lost), total cost (the total of direct and indirect cost), and average cost/effectiveness ratio of each treatment strategy will be calculated. Direct cost will include the cost of experimental drugs, laboratory and radiology examinations, AE/SAE treatment, traffic expenses and accommodation fees directly related with follow-up visit. Indirect cost will include loss of income due to the patients’ and their family’s inability to work due to RA. The incremental cost effectiveness ratio (ICER) of group A and group B against group C will be calculated. Cost-utility analysis will be performed by using EQ-5D-5L and SF-6D as utility index. The costs and average cost/utility ratio of each treatment strategy will be calculated. The incremental cost utility ratio [the cost of increase 1 quality adjusted life year (QALY)] of group A and group B against group C will be calculated. The treatment strategy will be considered to have cost-utility if the incremental cost utility ratio is below 3 times of China’s per capita GDP in the past year (¥100,000).

Non-parametric bootstrap will be used to calculate the 95% confidence interval of incremental cost effectiveness ratio and draw the cost-effectiveness acceptability curve. The factors which may affect the analysis results will be evaluated by one-way and probability sensitivity analysis. All the analysis will be performed by TreeAgePro11.0 software.

Safety analysis will be performed based on the safety set (SS). The incidence of AEs and SAEs related to the study will be described. Crosstabs will be constructed to map the changes of laboratory index.

## Discussion

The current study will be a multi-center, outcome assessment blinded, randomized clinical trial. The aim of this trial is to evaluate the efficacy, safety and cost-effectiveness of the triple therapy of csDMARDs (MTX + HCQ + SSZ) in reducing relapse risk compared with MTX monotherapy and TNFi plus MTX maintenance therapy in RA patients after achieving CR or LDA with TNFi plus MTX treatment. The triple therapy will be initiated 3 months before TNFi discontinuation. With this trial, we expect a better treatment strategy for RA patients who have achieved clinical target, to prevent relapse and simutaneously reduce cost.

In the past decades, some cost-effectiveness and cost utility studies associated with bDMARDs were performed on RA. A study from Australia discussed if it was worth ten years of publicly funded bDMARDs in the nation. However, it did not give guided recommendations [14]. A systematic review evaluated the cost-effectiveness of rituximab as first line for refractory RA patients comparing with csDMARDs, and found that rituximab was not cost-effective in Iran [15]. Hashemi et al. found that bDMARDs were cost-effective for RA patients based on a real-world setting in Japan [16]. Another study from Iran compared infliximab plus MTX with tocilizumab plus MTX in RA patients with inadequate response to csDMARDs and found that the regimen containing tocilizumab was not cost-effective comparing to the infliximab-containing regimen. [17]

The randomized Swedish Pharmacotherapy (SWEFOT) trial compared the infliximab + MTX and triple therapy in early RA patients with insufficient response to MTX. It reported a small but statistically significant difference in the radiographic outcome favoring the infliximab group, whereas disease activity, quality of life, and work loss improved similarly in both treatment arms, and no statistically significant difference in utility or QALY gain was detected [18–22]. But whether the triple therapy is as effective as bDMARDs in preventing relapse in RA patients who have achieved remission or LDA remains unclear.

Once approved, the study protocol may not be changed at will. In case the protocol must be verified, the principle investigator, the responsible person of clinical research center and the principle investigators at all sites should discuss and agree on the updates. When an agreement is achieved, protocol revision note should be formulated and signed, and the revised protocol should be submitted to the IRB for approval before implementation.

To guarantee the quality of the study, all the staffs should receive a standard training before start of the trial, including good clinical practice (GCP), subject protection and ethical requirements, study protocol, standard operation procedures, CRF filling instructions, and standard assessment method of disease activity. The project training will run throughout the whole process of the study. The investigators and the study coordinators can add training contents according to actual need.

Treatment that changes during the whole study period will be recorded. To guarantee the correct injection method, each injection of TNFi will be performed in the study centers by medical staffs rather than at home by patients themselves.

### Trial status

The current trial is ongoing and a total of 123 patients have been recruited into the second stage for randomized trial.

## Data Availability

The datasets generated and analyzed during the current study are available in the OpenClinica repository. The PERSISTENT WEB LINK TO DATASETS is http://202.112.183.17/bdfm/pages/login/login;jsessionid=C1AB75DF153AB5AD339AE4D1C560F88B.

## Abbreviations

RA: Rheumatoid arthritis
MTX: Methotrexate
bDMARDs: Biological disease-modifying anti-rheumatic drugs
TNFi: Tumor necrosis factor receptor Fc fusion protein
T-to-T: Treat to target
CR: Clinical remission
LDA: Low disease activity
EULAR: European League against Rheumatism
ACR: American Rheumatism Association
csDMARDs: Conventional synthetic disease-modifying anti-rheumatic drugs
HCQ: Hydroxychloroquine
SSZ: Sulafasalazine
DAS28: Defined as disease activity score 28
ICF: Informed consent form
IL-6: Interleukin-6
pSS: Primary SjÖgren syndrome
HIV: Human immuno-deficiency virus
ALT: Alanine aminotransferase
AST: Aspartate transaminase
PUCRI: Peking University Clinical Research Institute
IWRS: Interactive network answering system
NSAIDs: Non-steroidal antiinflammatory drugs
CRF: Case report form
CDAI: Clinical disease activity index
SDAI: Simplified disease activity index
HAQ-DI: Health Assessment Questionnaire Disability Index
EQ-5D-5L: The five-level Euro Qol five dimensions questionnaire
SF-6D: Short form-6D
PRO: Patient reported outcomes
AEs: Adverse events
SAE: Serious adverse event
IRB: Institutional Review Board
PV: Protocol violation
PUCRI-DM: Data Management Department of Peking University Clinical Research Institute
QALY: Quality-adjusted life-year
SS: Safety set
PUCRP: Peking University Clinical Research Institute Program Foundation

## References

[1] Katchamart W, Trudeau J, Phumethum V, Bombardier C (2009). Efficacy and toxicity of methotrexate (MTX) monotherapy versus MTX combination therapy with non-biological disease-modifying antirheumatic drugs in rheumatoid arthritis: a systematic review and meta-analysis. Ann Rheum Dis.

[2] Smolen JS, Landewé RBM, Bijlsma JWJ, Burmester GR, Dougados M, Kerschbaumer A (2020). EULAR recommendations for the management of rheumatoid arthritis with synthetic and biological disease-modifying antirheumatic drugs: 2019 update. Ann Rheum Dis.

[3] Smolen JS, Emery P, Fleischmann R (2014). Adjustment of therapy in rheumatoid arthritis on the basis of achievement of stable low disease activity with adalimumab plus methotrexate or methotrexate alone: the randomised controlled OPTIMA trial. Lancet.

[4] Migliore A, Bizzi E, Massafra U (2011). A new chance to maintain remission induced by anti-TNF agents in rheumatoid arthritis patients: CYnAR study II of a 12-month follow-up. Int J Immunopathol Pharmacol.

[5] Brocq O, Millasseau E, Albert C (2009). Effect of discontinuing TNFalpha antagonist therapy in patients with remission of rheumatoid arthritis. Joint Bone Spine.

[6] van den Broek M, Klarenbeek NB, Dirven L (2011). Discontinuation of infliximab and potential predictors of persistent low disease activity in patients with early rheumatoid arthritis and disease activity score-steered therapy: subanalysis of the BeSt study. Ann Rheum Dis.

[7] Navarro-Millán I, Sattui SE, Curtis JR (2013). Systematic review of tumor necrosis factor inhibitor discontinuation studies in rheumatoid arthritis. Clin Ther.

[8] Smolen JS, Nash P, Durez P (2013). Maintenance, reduction, or withdrawal of etanercept after treatment with etanercept and methotrexate in patients with moderate rheumatoid arthritis (PRESERVE): a randomized controlled trial. Lancet.

[9] Choy EHS, Smith C, Dore CJ, Scott DL (2005). A meta-analysis of the efficacy and toxicity of combining disease-modifying anti-rheumatic drugs in rheumatoid arthritis based on patient withdrawal. Rheumatology.

[10] O'Dell JR, Haire CE, Erikson N (1996). Treatment of rheumatoid arthritis with methotrexate alone, sulfasalazine and hydroxychloroquine, or a combination of all three medications. N Engl J Med.

[11] Soliman MM, Ashcroft DM, Watson KD (2011). Impact of concomitant use of DMARDs on the persistence with anti-TNF therapies in patients with rheumatoid arthritis: results from the British Society for Rheumatology Biologics Register. Ann Rheum Dis.

[12] Goekoop-Ruiterman YP, de Vries-Bouwstra JK, Allaart CF (2005). Clinical and radiographic outcomes of four different treatment strategies in patients with early rheumatoid arthritis (the BeSt study): a randomized, controlled trial. Arthritis Rheum.

[13] Hetland ML, Christensen IJ, Tarp U (2010). Direct comparison of Treatment responses, remission rates, and drug adherence in patients with rheumatoid arthritis treated with adalimumab, etanercept, or infliximab. Arthritis Rheum.

[14] Hopkins AM, Proudman SM, Vitry AI, Sorich MJ, Cleland LG, Wiese MD (2016). Ten years of publicly funded biological disease-modifying antirheumatic drugs in Australia. Med J Aust.

[15] Ahmadiani S, Nikfar S, Karimi S, Jamshidi AR, Akbari-Sari A, Kebriaeezadeh A. Rituximab as first choice for patients with refractoryrheumatoid arthritis: cost-effectiveness analysis in Iran based on a systematic review and meta-analysis. Rheumatol Int. 2016;36(9):1291–300. 10.1007/s00296-016-3484-5 27136919

[16] Tanaka E, Inoue E, Yamaguchi R (2017). Pharmacoeconomic analysis of biological disease modifying antirheumatic drugs in patients with rheumatoid arthritisbased on real-world data from the IORRA observational cohort study in Japan. Mod Rheumatol.

[17] Hashemi-Meshkini A, Nikfar S, Glaser E, Jamshidi A, Hosseini SA (2016). Cost-effectiveness analysis of tocilizumab in comparison with infliximab in iranian rheumatoid arthritis patients with inadequate response to tDMARDs: a multistage Markov model. Value Health Reg Issues.

[18] Eriksson JK, Karlsson JA, Bratt J, Petersson IF, van Vollenhoven RF, Ernestam S (2015). Cost-effectiveness of inflfliximab versus conventional combination treatment in methotrexate-refractory early rheumatoid arthritis: 2-year results of the register-enriched randomised controlled SWEFOT trial. Ann Rheum Dis.

[19] Eriksson JK, Neovius M, Bratt J, Petersson IF, van Vollenhoven RF, Geborek P, et al. Biological vs. conventional combination treatment and work loss in early rheumatoid arthritis: a randomized trial. JAMA Intern Med 2013;173:1407–14. 10.1001/jamainternmed.2013.7801 23817631

[20] Karlsson JA, Neovius M, Nilsson JA, Petersson IF, Bratt J, van Vollenhoven RF (2013). Addition of infliximab compared with addition of sulfasalazine and hydroxychloroquine to methotrexate in early rheumatoid arthritis: 2-year quality-oflife results of the randomised, controlled, SWEFOT trial. Ann Rheum Dis.

[21] Van Vollenhoven RF, Geborek P, Forslind K, Albertsson K, Ernestam S, Petersson IF (2012). Conventional combination treatment versus biological treatment in methotrexate-refractory early rheumatoid arthritis: 2 year followup of the randomised, nonblinded, parallel-group Swefot trial. Lancet.

[22] Bansback N, Phibbs CS, Sun H, O'Dell JR, Brophy M, Keystone EC (2017). Triple therapy versus biologic therapy for active rheumatoid arthritis: a cost-effectiveness analysis. Ann Intern Med.

